# Increased risk for diabetes development in subjects with large variation in total cholesterol levels in 2,827,950 Koreans: A nationwide population-based study

**DOI:** 10.1371/journal.pone.0176615

**Published:** 2017-05-18

**Authors:** Eun-Jung Rhee, Kyungdo Han, Seung-Hyun Ko, Kyung-Soo Ko, Won-Young Lee

**Affiliations:** 1 Department of Endocrinology and Metabolism, Kangbuk Samsung Hospital, Sungkyunkwan University School of Medicine, Seoul, Korea; 2 Department of Biostatics, The Catholic University of Korea, Seoul, Korea; 3 Division of Endocrinology and Metabolism, Department of Internal Medicine, St.Vincent’s Hospital, The Catholic University of Korea, Seoul, Korea; 4 Department of Internal Medicine, Cardiovascular and Metabolic Disease Center, Inje University Sanggye Paik Hospital, Inje University College of Medicine, Seoul, Korea; Shanghai Institute of Hypertension, CHINA

## Abstract

**Background:**

Recent studies suggest a role for hyperlipidemia in the development of diabetes. The aim of this study is to analyze the relationship between variations of total cholesterol (TC) levels and the risk for type 2 diabetes development from a Korean nationwide population-based database.

**Materials and methods:**

We examined the General Health Check-up sub-dataset of the Korean National Health Insurance Service (NHIS) of 2,827,950 participants who had at least three health check-ups between 2002 and 2007, and were not reported to have diabetes during that time. The variations of TC levels between the examinations were calculated as follows: $\text{TC}-\text{SD}=\surd \{\sum {\left({\text{x}}_{\text{i}}-\;\bar{\text{x}}\right)}^{2}/\left(\text{n}-1\right)\}$. The examinees were divided into 10 groups according to TC variation, and the hazard ratio for diabetes development from 2007 to 2013, were analyzed.

**Results:**

During the follow-up period, 3.4% of the participants had developed diabetes. The hazard ratio (HR) for diabetes development relative to the overall risk in the whole study population started to be higher than 1.0 from eighth decile of TC variation. The highest decile group showed an increased HR for diabetes development after adjustment for confounding variables (1.139; 95% confidence interval 1.116~1.163). These results were similar regardless of the use of anti-hyperlipidemic medication and baseline TC levels.

**Conclusions:**

The participants with a large variation in TC levels showed an increased risk for diabetes development, independent of the use of anti-hyperlipidemic medications. These results suggest a relationship between fluctuations in lipid levels and the development of type 2 diabetes.

## Introduction

According to the 7^th^ Diabetes Atlas released in 2015, 415 million people globally are suffering from diabetes [1]. More serious aspect is that the number will steadily increase in the future reaching 642 million patients worldwide in 2040. These observations are prominent in Asia-Pacific region, which contains two-thirds of world’s population [2].

Type 2 diabetes is known to be a strong determinant for cardiovascular disease, because hyperglycemia and insulin resistance cause atherosclerosis through chronic activation of the diacylglycerol-protein kinase C (DAG-PKC) pathway, which has been associated with vascular abnormalities [3]. Additionally, multiple risk factors for cardiovascular disease (CVD) are accompanied in patients with diabetes, such as dyslipidemia, hypertension and coagulopathy. Therefore, not only glucose control, but also multifactorial intervention for dyslipidemia and hypertension is needed to prevent vascular complications in patients with diabetes [4,5].

Dyslipidemia is an important risk factor that should be strictly controlled to prevent complications in patients with diabetes [6]. I*n vitro* and *in vivo* studies have shown that elevated extracellular and intracellular cholesterol levels lead to functional disturbances in pancreatic beta cells [7–9]. Clinical studies have shown that increased cholesterol levels lead to deterioration of glucose tolerance, and that a high total cholesterol (TC) to high-density lipoprotein cholesterol (HDL-C) ratio can predict type 2 diabetes [10,11].

However, a recent study has reported that patients with familial hypercholesterolemia, who have high levels of low-density lipoprotein cholesterol (LDL-C) due to genetic mutations in the function or expression of LDL receptors, have a lower risk for diabetes development [12]. In addition, a recent study has suggested an increased risk of diabetes development in participants taking 3-hydroxy-3-methylglutaryl-coenzyme (HMG-CoA) reductase inhibitors or statin [13]. Therefore, it is unclear whether low or high cholesterol levels affect the development of diabetes.

Despite the controversy regarding the relationship between serum cholesterol levels and diabetes development, there are no studies that have analyzed the influence of variation of lipid levels on diabetes risk. Therefore, in this study, we have analyzed the relationship between variation of serum cholesterol levels and the risk for diabetes in a nationwide population-based database (DB) of Korean residents.

## Methods

### Study population

This study is analyzed from the health check-up database (DB), which is a sub-dataset of Korean National Health Insurance Service (NHIS). The methods for utilization and analysis of this data were previously described [14,15]. In brief, the NHIS is a single-payer organization that is mandatory for all Korean residents. Because it has adopted a fee-for-service system to pay health care providers who treat or examine Korean patients, NHIS obtains information on patient demographics, medical use/transaction information, insurer’s payment coverage and patients’ deduction and claim database. NHIS database represent the entire Korean population; therefore, it can be used as a population-based, nationwide study for various diseases. Among sub-datasets of the NHIS database, we used only Health Check-up DB. This study was performed as one the 16 collaboration projects between Korean Diabetes Association (KDA) and NHIS. For general researchers who is not the collaborators of the 16 collaboration projects between KDA and NHIS, the data could be accessed after proposal submission to NHIS. The detailed information for data access of NHIS could be obtained from NHIS website (www.nhis.or.kr).

The Health Check-up DB generally consists of four areas: general health check-up, lifetime transition period health check-up, cancer check-up and baby/infant health check-up [1]. We used the general health check-up data, which includes (1) employee subscribers and regional insurance subscribers who are regional householders, (2) employee subscribers’ dependents and household members (40 years or older) and (3) Medical Aid beneficiaries who are either a householder (19 to 64 years) or a household member (41 to 64 years). All examinees were requested to have biannual health check-ups, except non-office workers who are employee subscribers (annual).

Among the general health check-up examinees, we selected 2,827,950 participants who had at least three examinations between 2002 and 2007. The fluctuation of total cholesterol level was analyzed in examinations between 2002 and 2006. Those with diabetes during the follow-up period and 2007 were excluded from the analysis. The medication history for anti-hyperlipidemic agents was only available in 2007. The development of diabetes was analyzed from the results of health check-up examinations from 2007 to 2013. A diagnosis of type 2 diabetes was defined by a fasting glucose level ≥126 mg/dL or a prescription for anti-diabetic medication with the presence of ICD-10 codes E11, E12, E13 or E14 [16].

The analyses presented in this paper were initially performed on all participants combined. We then performed sub-analyses based upon sex and the anti-hyperlipidemic medications the participants had been prescribed.

As this study was performed as one of the 16 projects in collaboration between Korean Diabetes Association (KDA) and NHIS [16], the review of the protocol was performed through the e-IRB system of Korea National Institute for Bioethics Policy site as the representative. Participants in National Health Check-up provided written informed consent for the use of their health screening data for research and only non-identified data with all the personal data were deleted were used for the analyses.

### Calculation of the TC variation

The mean value of serially measured cholesterol levels was calculated for each participant as the intra-individual mean (TC-mean). TC variability was measured as the standard deviation (SD) of serial TC measurements (TC-SD). The indices of TC variability were calculated as follows:

$$\text{TC}-\text{SD}=\;\sqrt{\frac{\underline{\sum {\left(x_{i}-\bar{x}\right)}^{2}}}{n-1}}$$

($\bar{x}$, mean of serially measured TC)

The distribution of the participants according to the number of measurements of TC is as follows: 44.4% of the participants measured twice, 17.3% measured three times, 15.1% measured four times and 23.2% measured 5 times.

The participants were divided into 10 groups according to the variations in TC levels. The mean variation of TC levels in each decile group was as follows: 4.36, 7.78, 10.26, 12.73, 15.00, 17.68, 20.82, 25.11 and 32.53%.

### Statistical analysis

Continuous variables are presented as the mean ± SD, the geometric mean (95% confidence interval [CI]) or the median (interquartile range). Categorical variables are presented as proportions. Statistical differences in the clinical characteristics between the participants who did and did not develop diabetes were determined by the student t-test or the Wilcoxon rank-sum test for continuous variables and the chi-squared test for categorical variables. The prevalence of diabetes among the decile groups was compared using a chi-squared test.

Cox-regression hazard model analyses were performed to analyze the risk for diabetes development in the highest decile group of TC variability. The analyses were repeated entering the TC-SD as a measure of TC variability. The results were expressed as hazard ratios (HRs) with 95% CI. In addition, the HR were expressed as the risk in deciles compared with the average risk in the whole study population. Kaplan-Meier survival analysis was used to draw the disease-free survival curve. In addition, similar analyses were performed with the HRs for diabetes development in group with TC-SD higher than or equal to 17.5 mg/dL, which is the population mean of TC variation.

Statistical analysis was performed using SAS version 9.3 (SAS Institute Inc., Cary, NC, USA). All p-values were two-sided, and a p-value <0.05 was considered significant.

## Results

### General characteristics of participants

The participant characteristics from 2007 are presented in Table 1. The mean age of the participants was 48 years. There were more men than women in this study. The mean body mass index (BMI) was 23.8 kg/m^2^ and more than half the participants were overweight.

**Table 1:** General characteristics of the participants according to the development of diabetes in 2007.

	Total	Development of diabetes		P value
		No	Yes	
N (%)	2,827,950	2,732,513(96.6)	95,437(3.4)	
Sex: men (%)	1,910,393(67.55)	1,848,780(67.66)	61,613(64.56)	<0.0011
Age (years)	48.11±11.08	47.9±11	54.7±11.4	<0.0011
Body mass index (kg/m^2^)	23.82±2.9	23.8±2.9	25.5±3.2	<0.0011
<18.5	64,787(2.29)	63,825(2.34)	962(1.01)	
18.5~23	1,052,781(37.23)	1,034,872(37.87)	17,909(18.77)	
23~25	778,058(27.51)	754,496(27.61)	23,562(24.69)	
25~30	861,975(30.48)	816,881(29.89)	45,094(47.25)	
>30	70,349(2.49)	62,439(2.29)	7,910(8.29)	
Systolic blood pressure (mmHg)	123.34±14.74	123.1±14.7	129.1±15.7	<0.0001
Diastolic blood pressure (mmHg)	77.45±9.95	77.3±9.9	80.4±10.3	<0.0001
Fasting blood glucose (mg/dL)	91.68±11.52	91.3±11.3	101.2±13.1	<0.0001
Total cholesterol (mg/dL)	195.57±35.42	195.2±35.2	205.6±38.9	<0.0001
Total cholesterol-standard deviation (mg/dL)	17.5±12.8	17.4±12.7	19.9±15.5	<0.0001
Proportion of subjects with total cholesterol≥ 240 mg/dL	308,432(10.91)	291,143(10.65)	17,289(18.12)	<0.0001
Hypertension (%)	461,471(16.32)	434,962(15.92)	26,509(27.78)	<0.0001
Current smoker (%)	765,058(27.97)	739,816(27.99)	25,242(27.44)	0.0002
Alcohol drinking ≥ 1 time per week (%)	895,197(32.38)	866,745(32.44)	28,452(30.62)	<0.0001
Exercise ≥ 3 times per week (%)	577,699(20.96)	557,112(20.91)	20,587(22.2)	<0.0001
Anti-hyperlipidemic medication (%)	153,153(5.42)	138,314(5.06)	14,839(15.55)	<0.0001
Anti-hypertensive medication (%)	385,427(13.63)	353,596(12.94)	31,831(33.35)	<0.0001

During the index period, 3.4% of the participants had developed diabetes (Table 1). The participants who had developed diabetes were older and more obese with more than half having a BMI higher than 25 kg/m^2^. Additionally, those that developed diabetes had higher mean blood pressures, fasting glucose levels and TC levels, and were more frequently taking anti-hyperlipidemic and anti-hypertensive medications. Mean TC-SD in total population was 17.5 mg/dL.

When the parameters were compared between men and women, women were older than men (51.8 vs. 46.3 years, S1 Table). The mean values of metabolic parameters such as lipid profiles were worse in women compared to men.

### Incidence rate for diabetes according to deciles of TC variation

**Fig 1:**
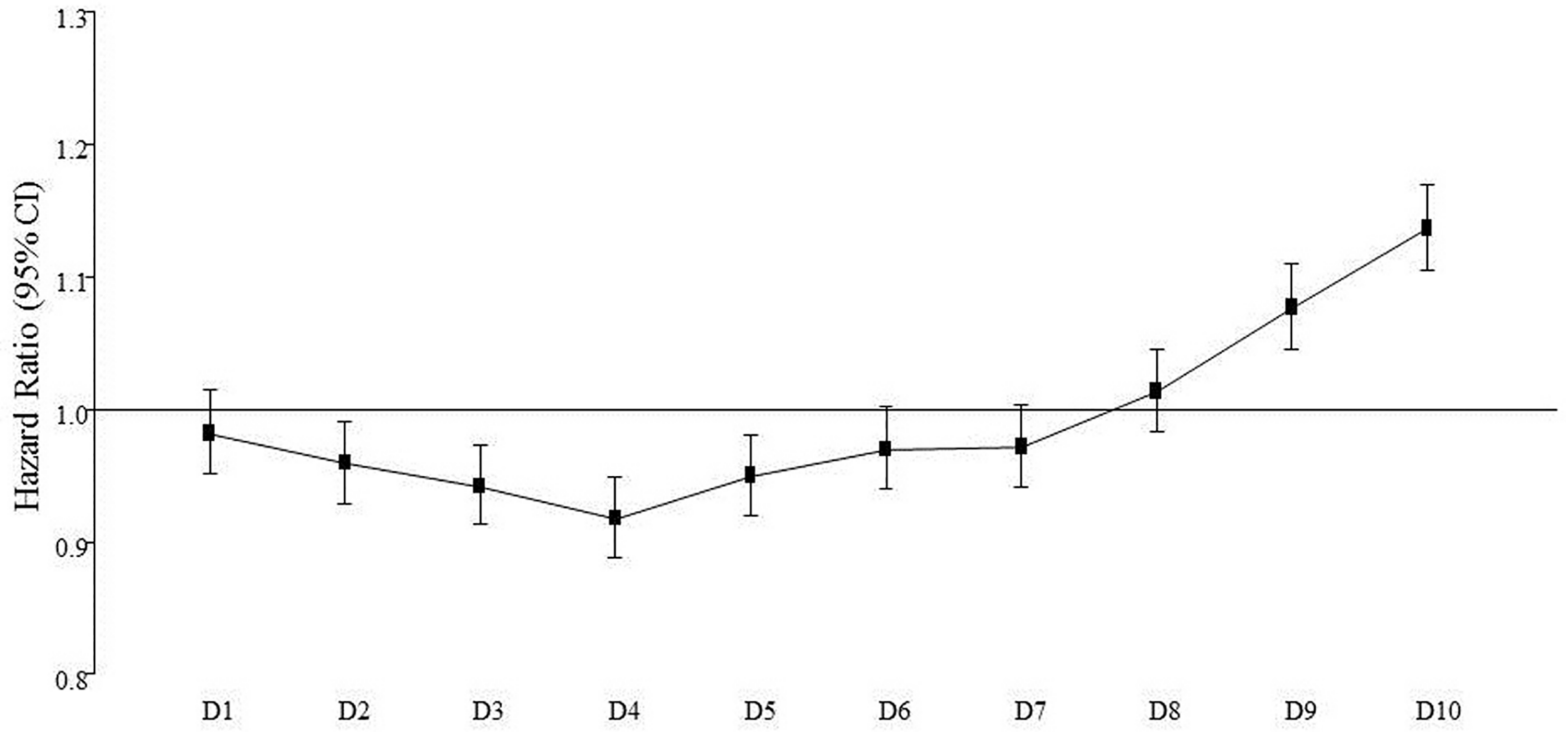
HR for diabetes development according to deciles of TC variations. The HR in this graph express the risk in deciles compared with the average risk in whole study population. HR, hazard ratio; CI, confidence interval; TC, total cholesterol.

When the incidence rate for diabetes and variation of TC levels were analyzed among the 10 groups, the participants with the highest decile of TC level variation had the highest incidence rate (cases per 100 person-year) for diabetes by 9.04 (S1 Fig). The number of participants and incident cases in each decile is shown on Supplemental S2 Table. The participants in the fourth decile of variation had the lowest incidence rate of diabetes by 4.61. In contrast, the diabetes incidence rates for the first to third deciles of variation were increased compared to the forth decile group. These trends were also observed when the participants were divided into two groups based upon their treatment with anti-hyperlipidemic medications (S2 Fig). However, when the HR for diabetes development were expressed as the risk in deciles compared with the average risk in the whole study population, HR for first to seventh decile of TC variation were lower than 1.0, and started to be higher than 1.0 from eighth decile of TC variation, continuously increasing (Fig 1).

**Fig 2:**
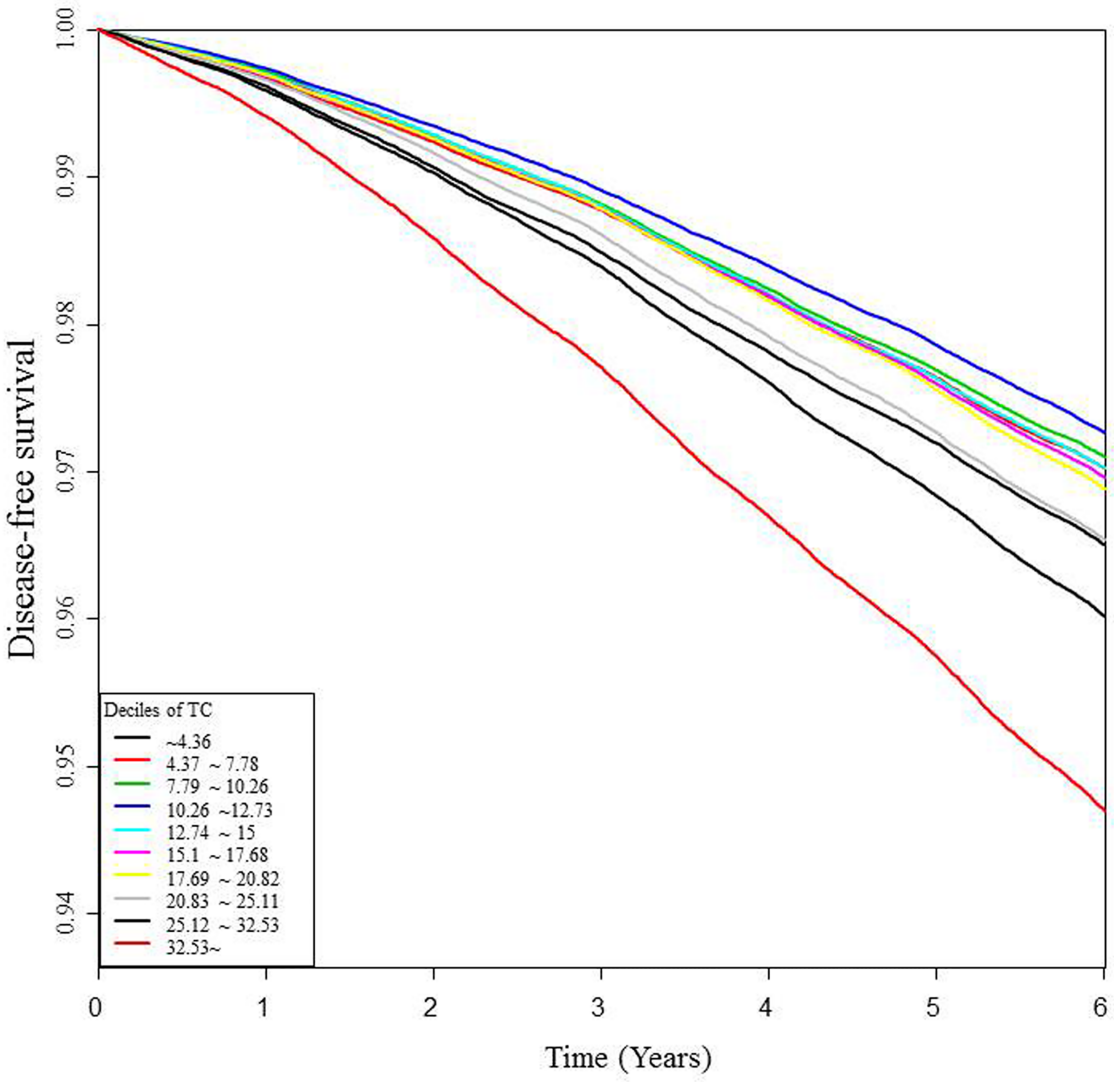
Disease-free survival curve for diabetes development according to the total cholesterol variation.

When Kaplan-Meier survival analyses were performed with diabetes development as the dependent variable, the group with the highest decile of variation showed the lowest disease-free survival rate for diabetes among the 10 decile groups of TC variation (Fig 2).

### Risk for diabetes development according to deciles of TC variation

The group with the highest decile of TC variation had a 1.601-fold increase in the HR for diabetes development compared with the first decile group of TC variation in crude analysis (Model 1 in Table 2). When age, sex, smoking, alcohol drinking, exercise and BMI were included in the model, the HR is attenuated to 1.282 (Model 2 in Table 2). When all variables were included in the model, the HR was attenuated to 1.139, which was still significant (Model 3 in Table 2). A high BMI (≥25 kg/m^2^), age (every 5 years increase), use of anti-hyperlipidemic medication, presence of hypertension and current smoking status showed a significantly increased risk for diabetes development. When this analysis was repeated with population mean of TC-SD as reference (<17.5 mg/dL), those with TC-SD higher than or equal to 17.5 mg/dL showed a HR of 1.107 of diabetes development after adjustment for confounding variables (S3 Table).

**Table 2:** Hazard ratio for development of diabetes after adjustment for confounding factors.

	Model 1	Model 2	Model 3
	HR (95% CI)	HR (95% CI)	HR (95% CI)
Variation of total cholesterol levels (highest decile vs. others)	1.601 (1.570,1.633)	1.282 (1.2579,1.309)	1.139 (1.116,1.163)
Age (every 5 years increase)		1.692 (1.683,1.702)	1.48 (1.471,1.489)
Sex: men		1.032 (1.015,1.048)	0.936 (0.921,0.951)
Fasting blood glucose (per 1 mg/dL increase)			1.061 (1.06,1.061)
Total cholesterol (per 1 mg/dL increase)			1.002 (1.002,1.003)
Anti-hyperlipidemic agent (yes)			1.634 (1.603,1.665)
Hypertension (yes)			1.404 (1.384,1.424)
Current smoker		1.383 (1.36,1.406)	1.437 (1.414,1.462)
Alcohol drinking (≥ 1 time per week)		0.983 (0.967,0.998)	0.872 (0.858,0.886)
Exercise (≥ 3 times per week)		0.965 (0.95,0.98)	0.968 (0.953,0.983)
Body mass index (kg/m^2^)			
<18.5		0.776 (0.726,0.83)	0.893 (0.836,0.955)
18.5–23		1.000 (reference)	1.000 (reference)
23–25		1.769 (1.734,1.804)	1.55 (1.519,1.582)
25–30		3.118 (3.063,3.174)	2.405 (2.361,2.449)
>30		7.397 (7.198,7.601)	4.763 (4.632,4.897)

HR, hazard ratio; CI, confidence interval

When analyses were performed for each sex, the trends were similar between men and women; however, men showed a higher risk for diabetes development in participants with the highest decile of TC variation even after adjustment for confounding variables (S4 Table). When this analysis was repeated with population mean of TC-SD as reference, men showed higher HR of 1.111 (95% CI 1.093~1.129) compared to women with 1.096 (95% CI 1.072~1.121) (S5 Table).

When we analyzed groups according to the use of anti-hyperlipidemic medications, the risk for diabetes development in the group with the highest decile of TC variation was more prominent for participants not taking anti-hyperlipidemic medications (HR 1.16; 95% CI 1.136~1.185 vs. 1.111; 95% CI 1.071~1.152; S6 Table). In participants who were taking anti-hyperlipidemic medications, the increased risk for diabetes development in the group with the highest decile of TC variation was significant, but markedly attenuated after adjustment for confounding variables. When the analysis was repeated with population mean of TC-SD as reference, those not taking anti-hyperlipidemic agent showed higher HR for diabetes development compared to those taking anti-hyperlipidemic agent (S7 Table).

## Discussion

In this study, the participants with the highest decile of TC variation showed the highest incidence rate for diabetes development during the follow-up period. The incidence rate for diabetes formed a J-shaped curve with the forth decile group showing the lowest incidence rate for diabetes development. The group with the highest decile of TC variation showed an increased risk for diabetes development after adjustment for confounding variables. These results were similar regardless of the use of anti-hyperlipidemic medications, suggesting that variations in TC levels contribute to the development of diabetes.

There are some previous studies on the association between hyperlipidemia and diabetes development. In a study performed in 5,577 Koreans without underlying diabetes, the TC to HDL-C ratio showed a significant association with an increased risk of type 2 diabetes over four years [10]. In a recent study by Wada et al. performed on 2,499 participants without diabetes, a high TC level was an independent risk factor for decreased insulin secretory capacity defined as a homeostasis model assessment index (HOMA)-beta ≤30% [11]. Additionally, another study showed that rat beta cell necrosis could be induced by prolonged exposure to LDL in a manner dependent on LDL cellular binding and internalization [17].

Mounting evidence indicates that elevated intracellular lipid metabolite concentrations are associated with diminished insulin sensitivity in both liver and skeletal muscle because excessive accumulation of toxic lipid metabolites, such as DAG and ceramide species, impair insulin action through the reduction of insulin-stimulated phosphorylation of insulin receptor substrate 2 (IRS2) and IRS2-associated phosphoinositide 3 (PI3) kinase activity [18]. There is numerous evidence that hypercholesterolemia itself could deteriorate glucose-stimulated insulin secretion and insulin sensitivity [7,19]. However, a recent study has suggested that patients with familial hypercholesterolemia with aberrantly high LDL-C levels have a significantly lower risk for diabetes development [12]. Therefore, there is a controversy over whether high or low cholesterol levels affect diabetes development.

In this study, participants with large variations in TC levels in at least two consecutive measurements showed a 1.16-fold increased risk for the development of diabetes. A plausible mechanism for the association of fluctuations of cholesterol level and diabetes development is unclear. However, both high and low cholesterol content in pancreatic islets from LDLR(-/-) and WT mice have shown decreased glucose-stimulated insulin secretion and Ca^2+^ handling, and normalization of cholesterol improved these abnormalities [20]. A study performed in an insulinoma tumor cell line also revealed that both high and low cellular cholesterol contents were inhibitory for insulin secretion [21]. Another study in rabbits has suggested that lipid fluctuation could accelerate the progression and vulnerability of atherosclerotic plaques through worsening arterial endothelial dysfunction and inflammatory reactions [22]. In line with the above mentioned studies, recent studies suggest that glycemic variability and visit-to-visit variability in BP could deteriorate pancreatic insulin secretory function and increase cardiovascular risk [23–27]. In addition, there is a possibility that the variability in cholesterol could have caused from insufficient adherence to anti-hyperlipidemic agents, although the results were similar in subjects with no anti-hyperlipidemic agents. It is well-known that adherence and persistence of statin are associated with reduction in CV risk and mortality [28]. From ours and from the previous results, we could assume that instability in metabolic parameters, such as glucose, BP or cholesterol levels could deteriorate insulin secretion and contribute to the development of atherosclerosis and diabetes. However, as our study was the first performed in humans, more studies are needed.

Our study has limitations. First, this was an observational study, not an interventional study. Therefore, the effects of confounding factors and bias could not be adjusted. However, the strength of our observational study is that it was performed on a large representative population. Second, there are many contributing factors that were not available in this DB, such as the type of anti-hyperlipidemic medications that the participants were taking. Third, the specific lifestyle factors of the participants, such as exercise, alcohol consumption and dietary patterns, could not be included in the analyses. Importantly, we could not include data on insulin levels in the analyses. Fourth, any changes in medication were not considered during the follow-up period. Fifth, the medication history for anti-hyperlipidemic agents from 2002 and 2006 were not considered in the analysis. However, as the results were similar whether they were taking anti-hyperlipidemic agens in 2007 or not, we could assume that not medication, but the fluctuation of TC level *per se* could have affects the outcome. In spite of these limitations, our studies offer a very novel and informative opinion on the relationship between lipid levels and diabetes development in this large Korean population.

In conclusion, we have shown for the first time that participants with a large TC level variation have an increased risk for diabetes development. These results were similar regardless of the use of anti-hyperlipidemic medications, suggesting that TC level variation affects the risk for diabetes development. Further studies are needed to clarify a plausible mechanism for the relationship between lipid fluctuation and type 2 diabetes development.

## Supporting information

S1 Fig The incidence rates for diabetes development according to TC level variations*(Cases per 1000 person-year). *Variations in TC level from the lowest to the highest deciles: 4.36, 7.78, 10.26, 12.73, 15.00, 17.68, 20.82, 25.11, and 32.53%.
(DOCX)

S2 Fig Proportion of subjects who developed diabetes in groups who were taking or not taking hyperlipidemic agent. (DOCX)

S1 Table Comparison of the parameters between men and women. (DOCX)

S2 Table The number of participants and incident cases in each decile. (DOCX)

S3 Table Hazard ratio for development of diabetes after adjustment for confounding factors using the population mean risk of TC-SD as reference. (DOCX)

S4 Table Hazard ratio (95% CI) for development of diabetes in different gender. (DOCX)

S5 Table Hazard ratio for development of diabetes after adjustment for confounding factors using the population mean risk of TC-SD as reference indifferent gender. (DOCX)

S6 Table Hazard ratio (95% CI) for development of diabetes according to hyperlipidemic agent. (DOCX)

S7 Table Hazard ratio for development of diabetes after adjustment for confounding factors using the population mean risk of TC-SD as reference according to hyperlipidemic agent. (DOCX)
