# Supplementary material for: Increased risk for diabetes development in subjects with large variation in total cholesterol levels in 2,827,950 Koreans: A nationwide population-based study
Source: PLoS One. 2017 May 18;12(5):e0176615. doi: 10.1371/journal.pone.0176615 (PMC5436642; doi:10.1371/journal.pone.0176615)
Supplement: S1 Fig — *Variations in TC level from the lowest to the highest deciles: 4.36, 7.78, 10.26, 12.73, 15.00, 17.68, 20.82, 25.11, and 32.53%. (DOCX) [file pone.0176615.s001.docx]

**S1 Fig.** The incidence rates for diabetes development according to TC level variations*(Cases per 1000 person-year). *Variations in TC level from the lowest to the highest deciles: 4.36, 7.78, 10.26, 12.73, 15.00, 17.68, 20.82, 25.11, and 32.53%.

**
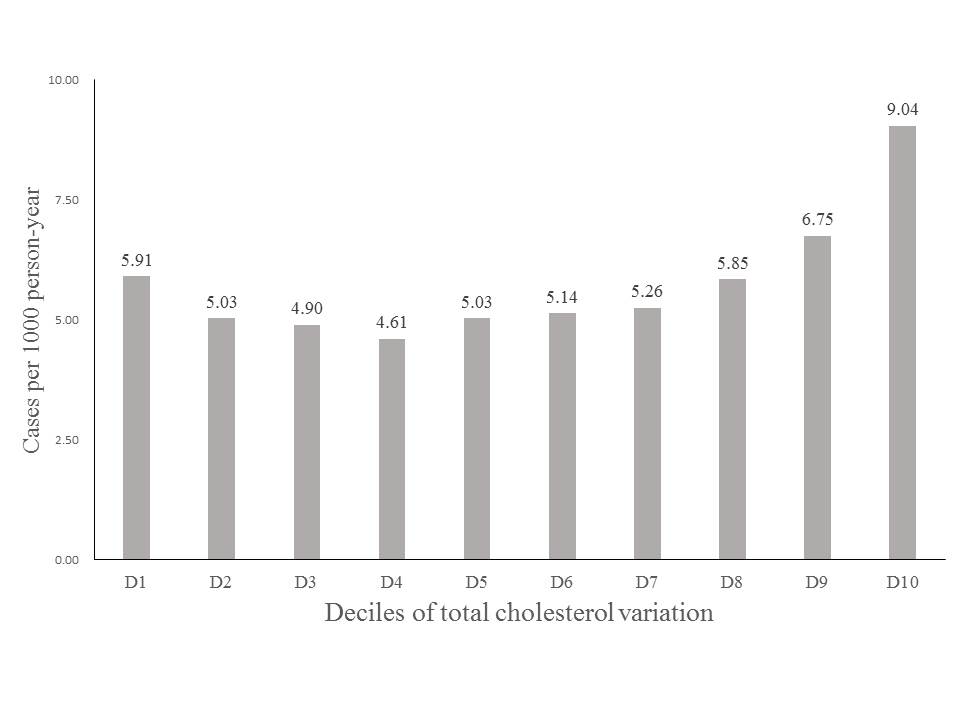
**
