# Supplementary material for: Increased risk for diabetes development in subjects with large variation in total cholesterol levels in 2,827,950 Koreans: A nationwide population-based study
Source: PLoS One. 2017 May 18;12(5):e0176615. doi: 10.1371/journal.pone.0176615 (PMC5436642; doi:10.1371/journal.pone.0176615)
Supplement: S2 Fig — (DOCX) [file pone.0176615.s002.docx]

**S2 Fig.** Proportion of subjects who developed diabetes in groups who were taking or not taking hyperlipidemic agent
