# Supplementary material for: Increased risk for diabetes development in subjects with large variation in total cholesterol levels in 2,827,950 Koreans: A nationwide population-based study
Source: PLoS One. 2017 May 18;12(5):e0176615. doi: 10.1371/journal.pone.0176615 (PMC5436642; doi:10.1371/journal.pone.0176615)
Supplement: S1 Table — (DOCX) [file pone.0176615.s003.docx]

**S1 Table.** Comparison of the parameters between men and women

|  | Men | Women | P value |
| --- | --- | --- | --- |
| N (%) | 1,910,393(67.55) | 917,557(32.45) |  |
| Age (years) | 46.33±10.73 | 51.81±10.87 | <0.0001 |
| Body mass index (kg/m^2^) | 24.03±2.84 | 23.36±2.99 | <0.0001 |
| <18.5 | 35,862(1.88) | 28,925(3.15) |  |
| 18.5~23 | 642,973(33.66) | 409,815(44.66) |  |
| 23~25 | 549,275(28.75) | 228,787(24.93) |  |
| 25~30 | 634,971(33.24) | 227,009(24.74) |  |
| >30 | 47,323(2.48) | 23,026(2.51) |  |
| Systolic blood pressure (mmHg) | 124.47±14.03 | 121±15.87 | <0.0001 |
| Diastolic blood pressure (mmHg) | 78.5±9.68 | 75.25±10.14 | <0.0001 |
| Fasting blood glucose (mg/dL) | 92.32±11.71 | 90.35±11 | <0.0001 |
| Total cholesterol (mg/dL) | 194.81±34.82 | 197.16±36.57 | <0.0001 |
| Total cholesterol-standard deviation (mg/dL) | 17.2±11.99 | 18.02±14.4 | <0.0001 |
| Proportion of subjects with total cholesterol≥ 240 mg/dL (%) | 291,143(10.65) | 17,289(18.12) | <0.0001 |
| Hypertension (%) | 328,134(17.18) | 133,344(14.53) | <0.0001 |
| Current smoker (%) | 753,033(40.55) | 12,031(1.37) | 0.0002 |
| Alcohol drinking ≥ 1 time per week (%) | 822,450(43.96) | 72,753(8.14) | <0.0001 |
| Exercise ≥ 3 times per week (%) | 402,300(21.59) | 175,402(19.64) | <0.0001 |
| Anti-hyperlipidemic medication (%) | 194,145(10.16) | 114,290(12.46) | <0.0001 |
| Anti-hypertensive medication (%) | 229,443(12.01) | 155,991(17) | <0.0001 |
