# Supplementary material for: Increased risk for diabetes development in subjects with large variation in total cholesterol levels in 2,827,950 Koreans: A nationwide population-based study
Source: PLoS One. 2017 May 18;12(5):e0176615. doi: 10.1371/journal.pone.0176615 (PMC5436642; doi:10.1371/journal.pone.0176615)
Supplement: S2 Table — (DOCX) [file pone.0176615.s004.docx]

**S2 Table.** The number of participants and incident cases in each decile

|  | Number of participants | Incident cases | Person-year | Incidence rate* |
| --- | --- | --- | --- | --- |
| Decile 1 | 284,567 | 9,767 | 1,651,869.99 | 5.91 |
| Decile 2 | 275,932 | 8,173 | 1,624,772.93 | 5.03 |
| Decile 3 | 289,525 | 8,362 | 1,706,196.67 | 4.90 |
| Decile 4 | 278,506 | 7,578 | 1,643,972.32 | 4.61 |
| Decile 5 | 286,846 | 8,500 | 1,690,168.97 | 5.03 |
| Decile 6 | 293,561 | 8,889 | 1,729,264.28 | 5.14 |
| Decile 7 | 271,402 | 8,403 | 1,598,464.76 | 5.26 |
| Decile 8 | 282,651 | 9,719 | 1,661,065.01 | 5.85 |
| Decile 9 | 278,302 | 11,006 | 1,629,939.5 | 6.75 |
| Decile 10 | 286,658 | 15,040 | 1,663,025.88 | 9.04 |
| Total | 2827950 | 95,437 | 16,614,424.19 | 5.74 |
| *Incidence per 1000 persons | |  |  |  |
