# Supplementary material for: Increased risk for diabetes development in subjects with large variation in total cholesterol levels in 2,827,950 Koreans: A nationwide population-based study
Source: PLoS One. 2017 May 18;12(5):e0176615. doi: 10.1371/journal.pone.0176615 (PMC5436642; doi:10.1371/journal.pone.0176615)
Supplement: S3 Table — (DOCX) [file pone.0176615.s005.docx]

**S3 Table.** Hazard ratio for development of diabetes after adjustment for confounding factors using the population mean risk of TC-SD as reference

|  | Model 1 | Model 2 | Model 3 |
| --- | --- | --- | --- |
|  | HR (95% CI) | HR (95% CI) | HR (95% CI) |
| TC-SD ≥ 17.5 mg/dL | 1.324(1.307,1.341) | 1.192(1.176,1.207) | 1.107(1.093,1.122) |
| Age (every 5 years increase) |  | 1.713(1.703,1.722) | 1.493(1.484,1.503) |
| Sex: men |  | 1.031(1.015,1.048) | 0.938(0.923,0.954) |
| Fasting blood glucose (per 1 mg/dL increase) |  |  | 1.061(1.06,1.061) |
| Total cholesterol (per 1 mg/dL increase) |  |  | 1.002(1.002,1.003) |
| Anti-hyperlipidemic agent (yes) |  |  | 1.644(1.613,1.675) |
| Hypertension (yes) |  |  | 1.405(1.385,1.426) |
| Current smoker |  | 1.387(1.364,1.411) | 1.443(1.419,1.467) |
| Alcohol drinking (≥ 1 time per week) |  | 0.981(0.966,0.996) | 0.869(0.856,0.883) |
| Exercise (≥ 3 times per week) |  | 0.963(0.948,0.978) | 0.965(0.95,0.981) |
| Body mass index (kg/m^2^) |  |  |  |
| <18.5 |  | 0.792(0.741,0.847) | 0.91(0.851,0.972) |
| 18.5-23 |  | 1.000 (reference) | 1.000 (reference) |
| 23-25 |  | 1.763(1.728,1.799) | 1.545(1.515,1.576) |
| 25-30 |  | 3.11(3.055,3.166) | 2.397(2.354,2.44) |
| >30 |  | 7.438(7.238,7.643) | 4.779(4.647,4.913) |
| TC-SD, total cholesterol-standard deviation; HR, hazard ratio; CI, confidence interval | | | |
