# Supplementary material for: Increased risk for diabetes development in subjects with large variation in total cholesterol levels in 2,827,950 Koreans: A nationwide population-based study
Source: PLoS One. 2017 May 18;12(5):e0176615. doi: 10.1371/journal.pone.0176615 (PMC5436642; doi:10.1371/journal.pone.0176615)
Supplement: S4 Table — (DOCX) [file pone.0176615.s006.docx]

**S4 Table.** Hazard ratio (95% CI) for development of diabetes in different gender

|  | Men | Women |
| --- | --- | --- |
| Variation of total cholesterol levels (highest decile vs. others) | 1.177 (1.15,1.206) | 1.126 (1.095,1.159) |
| Age (every 5 years increase) | 1.543 (1.531,1.554) | 1.363 (1.348,1.378) |
| Fasting blood glucose (per 1 mg/dL increase) | 1.058 (1.058,1.059) | 1.065 (1.064,1.066) |
| Total cholesterol (per 1 mg/dL increase) | 1.003 (1.003,1.003) | 1.002 (1.002,1.002) |
| Hyperlipidemic agent (yes) | 1.732 (1.689,1.776) | 1.526 (1.482,1.57) |
| Hypertension (yes) | 1.358 (1.334,1.382) | 1.501 (1.464,1.538) |
| Current smoker | 1.458 (1.432,1.483) | 1.539 (1.424,1.663) |
| Alcohol drinking (≥ 1 time per week) | 0.879 (0.864,0.893) | 0.846 (0.806,0.887) |
| Exercise ≥ 3 times per week | 0.947 (0.929,0.965) | 0.997 (0.97,1.024) |
| BMI (kg/m^2^) |  |  |
| <18.5 | 0.979 (0.902,1.063) | 0.752 (0.671,0.843) |
| 18.5-23 |  |  |
| 23-25 | 1.51 (1.472,1.548) | 1.671 (1.617,1.727) |
| 25-30 | 2.445 (2.39,2.502) | 2.427 (2.355,2.501) |
| 30- | 5.523 (5.331,5.721) | 3.976 (3.798,4.163) |
