# Supplementary material for: Increased risk for diabetes development in subjects with large variation in total cholesterol levels in 2,827,950 Koreans: A nationwide population-based study
Source: PLoS One. 2017 May 18;12(5):e0176615. doi: 10.1371/journal.pone.0176615 (PMC5436642; doi:10.1371/journal.pone.0176615)
Supplement: S5 Table — (DOCX) [file pone.0176615.s007.docx]

**S5 Table.** Hazard ratio for development of diabetes after adjustment for confounding factors using the population mean risk of TC-SD as reference indifferent gender

|  | Men | Women |
| --- | --- | --- |
| TC-SD ≥ 17.5 mg/dL | 1.111(1.093,1.129) | 1.096(1.072,1.121) |
| Age (every 5 years increase) | 1.559(1.548,1.571) | 1.371(1.356,1.386) |
| Fasting blood glucose (per 1 mg/dL increase) | 1.059(1.058,1.059) | 1.065(1.064,1.066) |
| Total cholesterol (per 1 mg/dL increase) | 1.003(1.003,1.003) | 1.002(1.002,1.002) |
| Hyperlipidemic agent (yes) | 1.749(1.706,1.794) | 1.529(1.485,1.573) |
| Hypertension (yes) | 1.358(1.335,1.383) | 1.504(1.467,1.541) |
| Current smoker | 1.464(1.438,1.489) | 1.552(1.437,1.678) |
| Alcohol drinking (≥ 1 time per week) | 0.876(0.862,0.891) | 0.844(0.805,0.885) |
| Exercise ≥ 3 times per week | 0.944(0.926,0.962) | 0.995(0.968,1.022) |
| Body mass index (kg/m^2^) |  |  |
| <18.5 | 1.005(0.925,1.091) | 0.759(0.677,0.851) |
| 18.5-23 | 1.000 (reference) | 1.000 (reference) |
| 23-25 | 1.504(1.467,1.543) | 1.667(1.613,1.723) |
| 25-30 | 2.437(2.382,2.494) | 2.421(2.35,2.495) |
| 30- | 5.547(5.354,5.746) | 3.992(3.813,4.179) |

TC-SD, total cholesterol-standard deviation; HR, hazard ratio; CI, confidence interval
