# Supplementary material for: Increased risk for diabetes development in subjects with large variation in total cholesterol levels in 2,827,950 Koreans: A nationwide population-based study
Source: PLoS One. 2017 May 18;12(5):e0176615. doi: 10.1371/journal.pone.0176615 (PMC5436642; doi:10.1371/journal.pone.0176615)
Supplement: S6 Table — (DOCX) [file pone.0176615.s008.docx]

**S6 Table.** Hazard ratio (95% CI) for development of diabetes according to hyperlipidemic agent

|  | Not taking hyperlipidemic agent in 2007 | Taking hyperlipidemic agent in 2007 |
| --- | --- | --- |
| Variation of total cholesterol levels (highest decile vs. others) | 1.16(1.136,1.185) | 1.111(1.071,1.152) |
| Age (every 5 years increase) | 1.53(1.52,1.54) | 1.198(1.177,1.22) |
| Sex(men) | 0.931(0.914,0.948) | 0.933(0.896,0.971) |
| Fasting blood glucose (per 1 mg/dL increase) | 1.062(1.061,1.063) | 1.054(1.052,1.055) |
| Total cholesterol (per 1 mg/dL) | 1.003(1.003,1.003) | 1.000(0.999,1.000) |
| Hypertension (yes) | 1.403(1.382,1.425) | 1.249(1.202,1.297) |
| Current smoker | 1.453(1.428,1.48) | 1.409(1.344,1.477) |
| Alcohol drinking (≥ 1 time per week) | 0.872(0.858,0.887) | 0.85(0.813,0.888) |
| Exercise ≥ 3 times per week | 0.968(0.951,0.985) | 0.952(0.916,0.989) |
| BMI (kg/m^2^) |  |  |
| <18.5 | 0.933(0.871,0.999) | 0.719(0.535,0.966) |
| 18.5-23 | 1 | 1 |
| 23-25 | 1.56(1.527,1.594) | 1.334(1.262,1.41) |
| 25-30 | 2.459(2.412,2.508) | 1.833(1.744,1.926) |
| 30- | 5.167(5.013,5.327) | 2.937(2.739,3.15) |
