# Supplementary material for: Increased risk for diabetes development in subjects with large variation in total cholesterol levels in 2,827,950 Koreans: A nationwide population-based study
Source: PLoS One. 2017 May 18;12(5):e0176615. doi: 10.1371/journal.pone.0176615 (PMC5436642; doi:10.1371/journal.pone.0176615)
Supplement: S7 Table — (DOCX) [file pone.0176615.s009.docx]

**S7 Table.** Hazard ratio for development of diabetes after adjustment for confounding factors using the population mean risk of TC-SD as reference according to hyperlipidemic agent

|  | Not taking hyperlipidemic agent in 2007 | Taking hyperlipidemic agent in 2007 |
| --- | --- | --- |
| TC-SD ≥ 17.5 mg/dL | 1.101(1.085,1.117) | 1.075(1.039,1.112) |
| Age (every 5 years increase) | 1.533(1.523,1.543) | 1.201(1.18,1.222) |
| Sex(men) | 0.927(0.911,0.944) | 0.93(0.893,0.969) |
| Fasting blood glucose (per 1 mg/dL increase) | 1.062(1.061,1.063) | 1.054(1.052,1.055) |
| Total cholesterol (per 1 mg/dL) | 1.003(1.003,1.003) | 1(0.999,1) |
| Hypertension (yes) | 1.403(1.382,1.425) | 1.249(1.202,1.297) |
| Current smoker | 1.453(1.428,1.48) | 1.409(1.344,1.477) |
| Alcohol drinking (≥ 1 time per week) | 0.872(0.857,0.887) | 0.849(0.813,0.887) |
| Exercise ≥ 3 times per week | 0.968(0.951,0.984) | 0.953(0.917,0.99) |
| BMI (kg/m^2^) |  |  |
| <18.5 | 0.934(0.872,1.001) | 0.72(0.536,0.967) |
| 18.5-23 | 1.000 (reference) | 1.000 (reference) |
| 23-25 | 1.559(1.526,1.593) | 1.335(1.263,1.411) |
| 25-30 | 2.458(2.411,2.507) | 1.834(1.745,1.928) |
| 30- | 5.167(5.012,5.326) | 2.94(2.742,3.153) |
